# Mounting evidence that managed and introduced bees have negative impacts on wild bees: an updated review

**DOI:** 10.1016/j.cris.2022.100043

**Published:** 2022-07-22

**Authors:** Jay M. Iwasaki, Katja Hogendoorn

**Affiliations:** School of Agriculture, Food and Wine, The University of Adelaide, Adelaide SA 5064, Australia

**Keywords:** Apis, Bombus, Competition, Managed, Bees, Pollinators, Pathogens

## Abstract

Worldwide, the use of managed bees for crop pollination and honey production has increased dramatically. Concerns about the pressures of these increases on native ecosystems has resulted in a recent expansion in the literature on this subject. To collate and update current knowledge, we performed a systematic review of the literature on the effects of managed and introduced bees on native ecosystems, focusing on the effects on wild bees. To enable comparison over time, we used the same search terms and focused on the same impacts as earlier reviews. This review covers: (a) interference and resource competition between introduced or managed bees and native bees; (b) effects of introduced or managed bees on pollination of native plants and weeds; and (c) transmission and infectivity of pathogens; and classifies effects into positive, negative, or neutral. Compared to a 2017 review, we found that the number of papers on this issue has increased by 47%. The highest increase was seen in papers on pathogen spill-over, but in the last five years considerable additional information about competition between managed and wild bees has also become available. Records of negative effects have increased from 53% of papers reporting negative effects in 2017 to 66% at present. The majority of these studies investigated effects on visitation and foraging behaviour. While only a few studies experimentally assessed impacts on wild bee reproductive output, 78% of these demonstrated negative effects. Plant composition and pollination was negatively affected in 7% of studies, and 79% of studies on pathogens reported potential negative effects of managed or introduced bees on wild bees. Taken together, the evidence increasingly suggests that managed and introduced bees negatively affect wild bees, and this knowledge should inform actions to prevent further harm to native ecosystems.

## Highlights

• Worldwide there is increasing reliance and concern for pollinators, primarily bees
• Wild bees may be threatened by habitat loss, pathogens, and perhaps managed bees
• The literature supports significant evidence for competition from managed bees
• However, the factors affecting bee competition need be examined in context
• Conserving pollinators requires action that focuses less on managed species

## Introduction

In the last two decades, an increasing number of studies have reported local and global declines in the diversity and abundance of wild bees (Biesmeijer et al., 2006; Burkle et al., 2013; Colla and Packer, 2008; Dicks et al., 2016; Koh et al., 2016; Potts et al., 2010; Senapathi et al., 2015; Zattara and Aizen, 2021). The drivers of these declines are various, synergistic, and can differ between continents and regions (Dicks et al., 2021). Declines can in some cases be linked to climate change (Forrest, 2015; Hegland et al., 2009), spread of pathogens (Cameron et al., 2011), introduction of non-native plant and pollinator species, and pesticide use (Goulson et al., 2015; Woodcock et al., 2016). However, the most important driver for native bee declines is thought to be loss of habitat, both for nesting and floral resources brought on by land-use and land cover changes (Dicks et al., 2021; Kennedy et al., 2013; Kremen et al., 2002; Tscharntke et al., 2005). Decreases in density and diversity of pollen sources for wild bees in the landscape are a consequence of clearing of wild bee habitat, urbanisation, agricultural intensification, and loss of farmland heterogeneity (Herbertsson et al., 2021; Winfree et al., 2011). Observations of declines in wild bee and insect-pollinated plant species along gradients of land-use intensity and agricultural intensification support these inferred causal factors (Clough et al., 2014; Gabriel and Tscharntke, 2007).

In contrast to the decline of wild bees, managed bees, including honey bees and certain species of bumblebees, are on the increase (Aizen et al., 2022; Aizen and Harder, 2009; Osterman et al., 2021). Honey bees have been introduced in nearly all countries in the world (Osterman et al., 2021). In some areas, the proportion of wild bees to honey bees documented on flowers has shifted substantially towards honey bees over the last 50 years (Herrera, 2020). The increase in managed hives is partly caused by a substantial increase in demand for crop pollination, which is a result of both an absolute and relative increase in the area used to produce pollination dependent crops (Aizen et al., 2022, 2008; Aizen and Harder, 2009). Wild bees can be abundant pollinators in agricultural landscapes with landscape heterogeneity, however simplified agricultural landscapes may lead to reductions in wild bee populations, further contributing to increased demand for managed hives for pollination (Hass et al., 2018; Kennedy et al., 2013; Winfree et al., 2008).

The main species of managed bees are honey bees (*Apis mellifera*; Osterman et al., 2021), bumblebees (genus *Bombus*, specifically *B. terrestris* and *B. impatiens*) and the solitary alfalfa leafcutter bee (*Megachile rotundata*). While bumblebees are largely managed for crop pollination, the honey bee industry relies only partly on crop pollination for its revenue. A larger part of the income is derived from honey production (e.g., approximately 50% in the USA and 80% in Australia; Clarke and Le Feuvre, 2021; Ferrier et al., 2018). Global honey consumption, demand and price have steadily increased over the past decades (García, 2018). Honey is preferentially produced from natural vegetation, as these honeys often fetch higher prices than those produced from crops. In addition, in contrast to bumblebee colonies, honey bee hives used for pollination are maintained when not deployed to pollinate crops and require pollen and carbohydrates as food. Native vegetation is preferred over artificial feeding or cropping areas because this requires less maintenance, lowers costs, and provides more diverse floral resources in the absence of pesticides. Furthermore, honey bee collected pollen is used to breed managed bumblebees for crop pollination.

The increase in use of managed bees, reduced floral support, high pesticide use in agricultural landscapes, and increasing demand for pollen and honey produced from native vegetation and wildflowers, work hand in hand to increase the demand of the beekeeping industry for access to native vegetation on public and private land. Concern is rising that this may put additional stress on many of the more than 20,000 native wild bee species and their important pollination services, both in cropping environments and natural vegetation (Aizen and Harder, 2009; Angelella et al., 2021; Osterman et al., 2021; Rasmussen et al., 2021; Winfree et al., 2008).

In agricultural areas, diverse wild bee communities have been found to increase pollination and subsequent crop yields even when managed bees are present (Garibaldi et al., 2013; Hoehn et al., 2008; Winfree et al., 2008). However, the increase in managed hives, together with the decrease in abundance and diversity of floral resources, which traditionally supports bee diversity (e.g., Decourtye et al., 2010; Hopwood, 2008), may increase resource competition between wild and managed bees in cropping areas (Angelella et al., 2021; Osterman et al., 2021). If this is the case, there is potential for a vicious cycle, where an increased use of managed bees leads to lower levels of floral resources for wild crop pollinating bees, which decreases their abundance and pollination services, which in turn leads to an even greater demand for managed hives.

There are several reasons why this would be an undesirable outcome. First, dependence on a single species for pollination is risky, as there is no resilience in case of disease outbreak and the high density use of a single species makes such outbreaks more likely (Gisder and Genersch, 2017). Second, it provides an industry with a monopoly on an important service. Third, it would affect other flying insects which require nectar and pollen. Wild bees are not the only animals that depend on floral resources in the landscape. Low densities of flying insects in agricultural landscapes not only reduce pollination services, but also biological control and the amount of food for vertebrates (Bianchi and Wäckers, 2008; Bowler et al., 2019; Raven and Wagner, 2021; Siekmann et al., 2004). In many natural habitats, a diverse wild bee community is integral for maintaining plant diversity and ecosystem function (Fontaine et al., 2006; Memmott et al., 2004). Therefore, when managed hives of social species with many individuals and high foraging needs and efficiencies are allowed access to natural environments either to increase hive strength, or to collect honey for human consumption or pollen for managed bee breeding enterprises, resource competition can result in negative impacts for wild bee communities and their pollination services to native vegetation (Cane and Tepedino, 2017; Henry and Rodet, 2018). In particular, in countries where honey bees are not native, one could argue that in conservation areas, ecosystems should be protected against such pressures (Wojcik et al., 2018).

Several reviews of the interactions between managed and introduced bees and native bees have been produced in the last 20 years (Geslin et al., 2017; Goulson, 2003; Mallinger et al., 2017; Paini, 2004; Wojcik et al., 2018). These reviews increasingly suggest that managed honey bees and bumblebees can have substantial impacts on wild bee fitness as a result of resource competition. However, as noted by the reviewers, most studies are correlative and demonstrate circumstantial evidence for competition between managed and wild bees including effects on various aspects of native bee foraging behaviour. Unsurprisingly, such correlative methods yield equivocal results, as they only document the potential for impact, but do not experimentally assess the direct effects on wild bee fitness, abundance, or diversity. Absence of observable effects in such studies can be a consequence of permanent changes in bee composition, floral composition, or even foraging behaviour due to past competition.

Experimental studies can also yield equivocal outcomes (Wojcik et al., 2018). In particular, artificial increases in managed bees may not necessarily result in changes in densities of local native bee species on flowers. A reduction in floral resource availability, as a result of an experimental increase in managed bees, could increase rather than decrease the time other bee species spend foraging (Thomson, 2004). However, the increased competition would then be expected to come at a cost of foraging returns and energy inputs into reproduction. The fact that this has been found to be the case (Thomson, 2004, 2006) demonstrates that the metrics of correlative and experimental studies should be carefully chosen (Wojcik et al., 2018). In addition, more experimental studies are urgently needed. Of the seven studies that experimentally investigated the effects of honey bee densities on either colony growth (*Bombus* spp.) or reproductive outputs (*Bombus* spp. and various solitary bee species), six indicated negative effects (Wojcik et al., 2018).

Given the rapidly increasing number of studies on the effects of managed and introduced bees on natural ecosystems and the escalating dependency on managed bees, up to date information is critical for informing management decisions. The concomitant decline of wild bees, their importance for ecosystem functioning, as well as the increased pressures on natural environments in general, makes it essential to closely monitor further developments regarding the influence of managed hives on the health of native ecosystems. In this paper, we present a systematic review of the current body of literature that examines the effects of managed and introduced bees on native ecosystems through resource and displacement competition with wild pollinators, through changes in plant-pollinator networks, and through transmission of pathogens. Compared to the most recent review on this topic (Mallinger et al., 2017), our study includes roughly 50% more publications, which reflects a rapidly growing body of literature. In addition, our review expands the information available for specific categories and summarises reported outcomes for interspecific interactions. This information should help to better target future research on pollinator conservation, inform land managers about the risks of managed hives to native bee communities, and highlights the importance of providing floral support for bees in agricultural landscapes.

## Methods

**Fig. 1:**
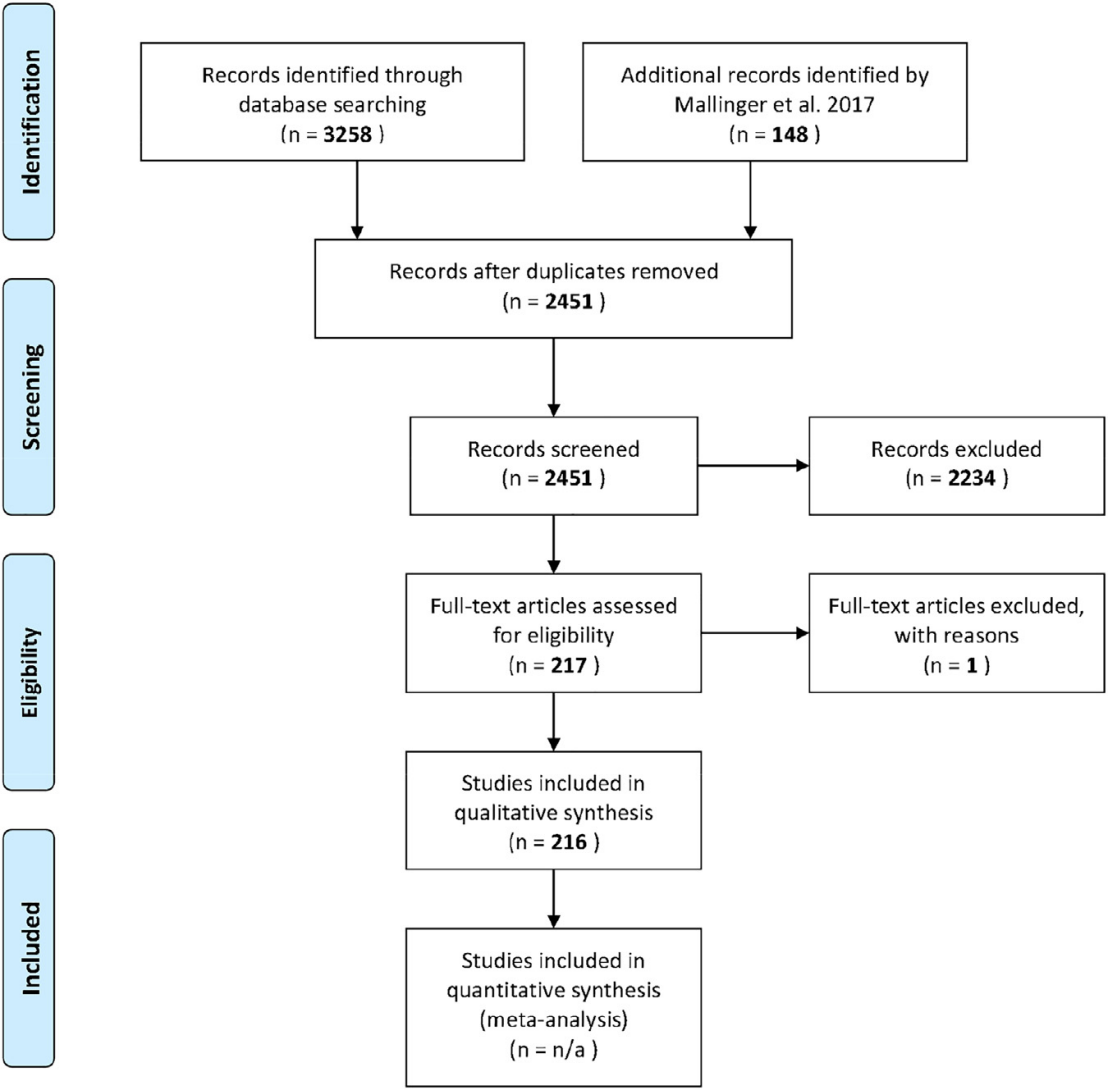
**PRISMA flow diagram.** A flow diagram depicting the steps for a systematic literature review following the steps outlined by the Preferred Reporting Items for Systematic Reviews and Meta-Analyses (PRISMA) statement. Each box shows the number of studies included after each step in the process.

Our review updated and expanded from the body of literature reviewed by Mallinger et al. (2017). Both Mallinger et al. (2017) and our review are based on the guidelines of Preferred Reporting Items for Systematic Reviews and Meta-Analyses (PRISMA; Moher et al., 2009), which allows for transparency and reproducibility in each step for subsequent reviews (PRISMA flow diagram, Fig. 1). The objectives of the two reviews were similar: to identify studies that examine the impacts of managed and introduced bees on native species of plant and animals through changes in pollination networks, competition, and transmission of pathogens, and summarise the current body of literature.

To conduct our review, we first assembled the dataset of 148 articles that were included in the quantitative analysis published by Mallinger et al. (2017). We then conducted a systematic search for their time period (up to 2017) of the literature using Web of Knowledge/Web of Science (ISI Thompson-Reuters, webofknowledge.com) using the exact same terms (“Apis mellifera” OR “honey bee” OR honeybee) AND (competition OR disease OR pathogen OR (pollin* AND (exotic OR invasive))), as well as the same with each of their subsequent specified terms: “managed bee” AND (competition OR disease OR pathogen OR (pollin* AND (exotic OR invasive))). Our search methods found the exact results as reported, confirming our search terms were congruous. We then updated search dates for 2017 onwards, and conducted additional checks of the two review articles cited by Mallinger (i.e., Goulson, 2003; Paini, 2004) and all recent articles published after 2016, citing those same two reviews. The literature search was completed in August 2021. We used similar metrics as Mallinger at al. (e.g., bee/floral visitors, location, context, managed bee metrics, explanatory mechanism variables, wild bee metric, reported effect) and tallies of reported outcomes. In addition, expanded information was gathered by including additional metrics and notes. Specifically, we included more detail on impacted species, bee sizes where available, general and specific taxonomic groups, apiary additions, observational or manipulative studies, competition and competition types, summaries in detail, and additional notes for context.

To maintain continuity for subsequent reviews, we followed the same evaluation methods as Mallinger et al. (2017). Articles were selected based on whether they included the impacts of an introduced or managed bee species on native floral visitors, plant communities, and disease dynamics. We specifically included all newer studies pertaining to the honey bee (*Apis mellifera*) because it was not always possible to differentiate whether they were native, feral, or originating from managed hives in studies within their native range when observing foraging honey bees (Africa and Eurasia). Where authors were able to differentiate their origin, we specified that information. Papers that did not include impacts on another species were not included.

We included only papers that were peer-reviewed, in English, and not part of grey literature. Studies were required to have a reported outcome effect that could be measured. For honey bees, this filtering reduced an initial 1593 articles to 1373; for the bumblebees, an initial 944 reduced to 807; and for managed bees, filtering reduced 866 articles to 721. After manually screening all records using these specific criteria, this resulted in 69 new studies published after 2016. Adding these to Mallinger's 148 papers results in a total of 217 studies included in the quantitative synthesis. One of the 148 articles was removed due to relevance for a total of 216 (Fig. 1).

We used only inferences made by authors regarding the effect of the managed/introduced species on the subject under investigation. These effects were classified as either negative (-1), no effect (0), positive (1), or potentially negative (-0.5). Potentially negative scores indicate that, based on the data, the authors suggested potential harm in the discussion. This happens for example, when, based on observations of resource overlap and shortage of resources, the authors inferred potential negative consequences for the reproductive output of the subject under investigation. Effects were scored in regards to their ecological context. For example, negative scores for effects of managed/introduced bee species on plant species can refer to increased seed production of an invasive plant species, or to a decreased seed production of a native plant species. No effect (0) suggests a non-significant effect. Positive effects (1) suggest that there was some benefit of the presence of the managed/introduced species for the native species studied. In the context of pathogens, harm means that pathogen prevalence had or had demonstrated potential to either increase, or that the pathogen would cause a negative impact on fitness, abundance, or diversity of the focal group. No effect in the context of pathogens implies that there was no measurable effect on pathogens prevalence. Notably, there were no positive outcomes associated with pathogen studies. Scores were tallied for each category of interest (bee competition, plant interaction, pathogen effects) and discussed together. These criteria followed the same concepts as in Mallinger et al. (2017) to ensure uniformity in results and conclusions. Similarly, we decided against conducting meta-analyses because of the wide variety of metrics associated with these studies, which makes standardising criteria very difficult.

In addition, we briefly investigated the importance of bee sizes for competition. Henry and Rodet (2018) suggest that large sized bees are more impacted by competition from managed honey bees than small sized bees. To further explore this, we included body length, based on species descriptions, where specific bee species were mentioned. For species with a range of sizes (e.g., workers of *Bombus* spp.), we used the median length to reflect the sizes of foragers when competition is most likely to be occurring. We then classified these sizes as smaller, similar and larger than honey bee workers (see Table 2), and discussed the impact on the different size classes.

## Results and discussion

**Fig. 2:**
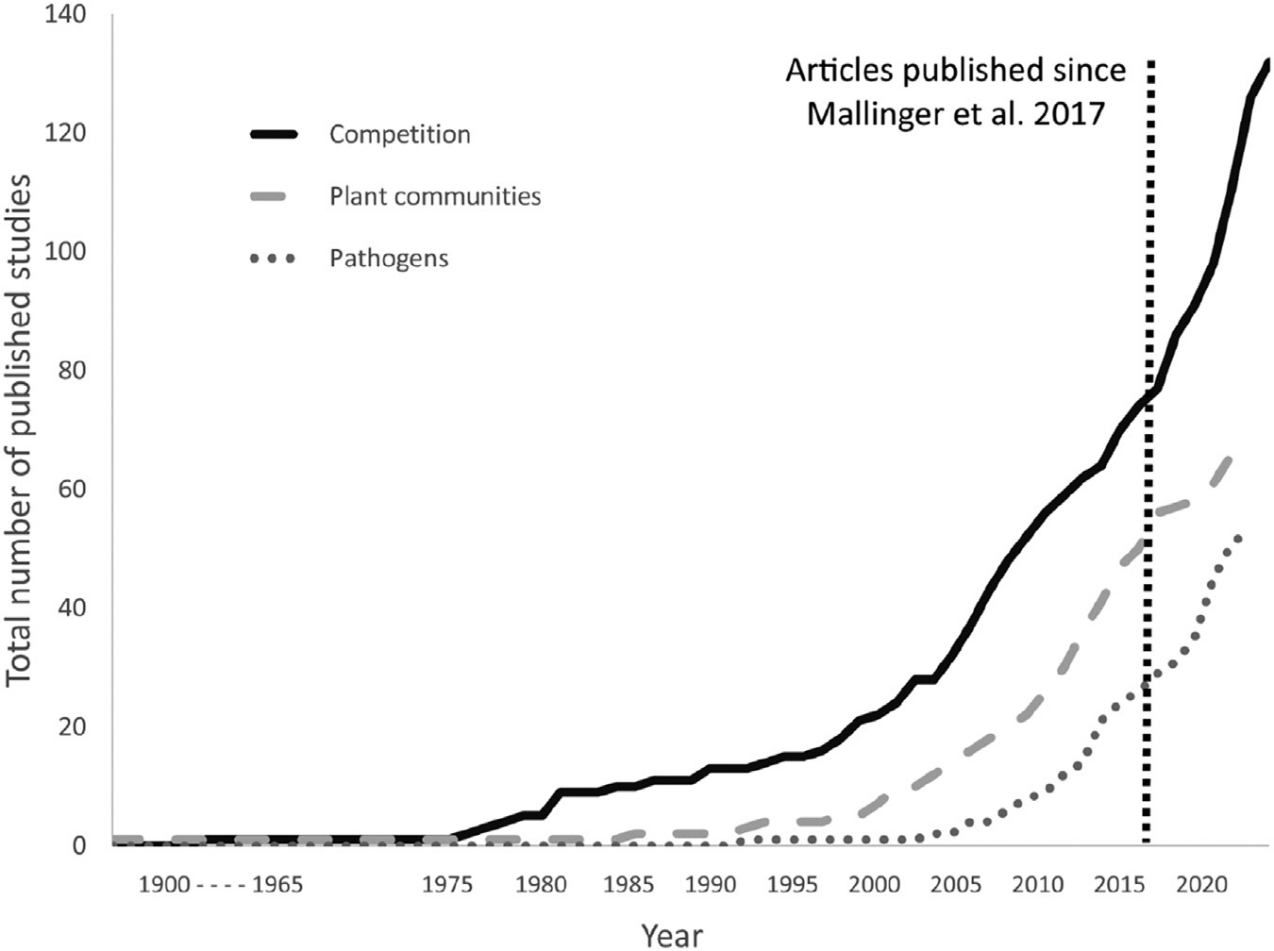
**Growth in publication trends.** The cumulative number of studies from 1900 – 2021. Publications were classified into three categories: studies examining (a) competition between bee species; (b) changes in plant communities through bee interactions; and (c) effects through bee pathogen transmission. The vertical dotted line refers to the last literature review by Mallinger et al. (2017) to compare the growth of each corresponding category.

Our search settings yielded 216 articles, an increase of 69 articles (+47%) compared to the last similar review in 2017 (Fig. 2). The papers included 229 outcomes. Of these outcomes, 95 specifically involved competition between bees, 74 involved effects on plants/pollination networks, and 58 focused on pathogens/disease. Single additional studies considered the impacts on bird nesting and modelling potential contributors to competition. Cumulatively, the increase in published research on this topic followed established trends, showing gradual increases in the number of papers focusing on competition, but relatively greater increases in the number of studies focusing on disease and pollination (see Mallinger et al. 2017, Fig. 3). In comparison to the other categories, the proportional increase in the number of studies pertaining to interactions that included pathogens was highest of all categories (+45%), reflecting consistent trends in research interest.

**Fig. 3:**
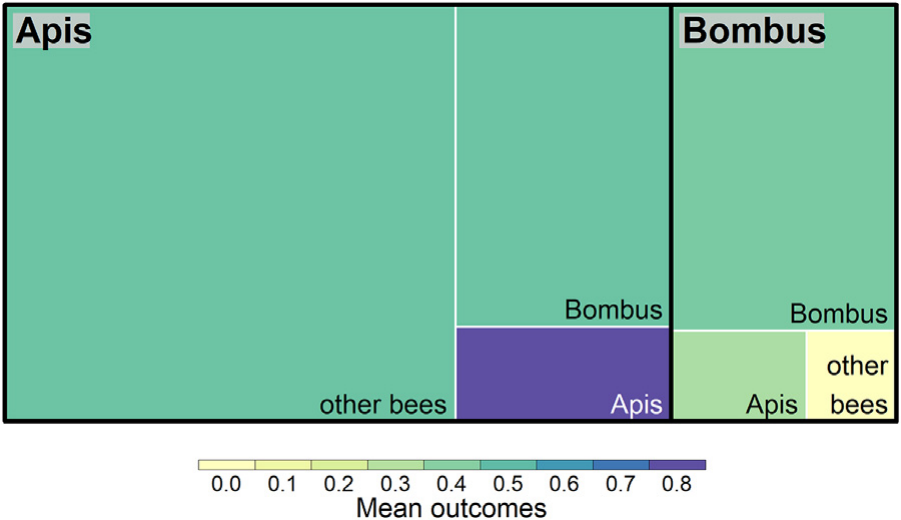
**Impacts of bee genera on other bee groups.** A treemap of study outcomes that assessed competition from bees in the genus *Apis* and *Bombus* against other bee genera/groups (n = 91). The size of each box indicates the respective number of studies, and the colour represents the mean harmfulness of all outcomes for each category where 0 is harmless and 1 is harmful. Of the 68 *Apis* studies, 65 involved *Apis mellifera,* and of 23 *Bombus* studies, 18 involved to *Bombus terrestris* as managed/introduced species. Only two studies explored the impacts of other bee groups on *Bombus* (n = 1, no harm) or other bees (n = 1, harmful). These were excluded from this diagram.

As is typical in reviews on bees or pollinators, studies involving honey bees (*Apis mellifera*) dominate the literature (Arena and Sgolastra, 2014; Goulson, 2003; Iwasaki and Hogendoorn, 2021a; Mallinger et al., 2017), followed by bumblebees (*Bombus* spp.). Studies with interactions that involved *Apis* composed 78% (n = 178) of all study outcomes. Studies that focused on species of *Bombus* (n = 71) involved 31% of outcomes.

### Geographic and temporal trends in research interest

Similar to other reviews (Dicks et al., 2021; Goulson, 2003; Mallinger et al., 2017; Paini, 2004), studies from North America and Europe dominated publications on bee competition. With respect to other regions, states in Oceania (Australia, New Zealand, New Caledonia, Hawaii, Pacific nations) and South America had significant representation, east and central Asian countries were under-represented, and Africa was the most under-represented continent. Overall compared to 2017, the greatest increase in the number of studies were in Oceania, North America, and Europe. Increases in the number of studies involving effects on pollination came primarily from Europe, and these mostly related to the impacts of honey bees. The increase in studies on pathogen transmission and infectivity was largely due to research undertaken in Europe and South America. Most of these related to recently introduced bumblebees in Argentina and Chile, as well as the impacts of honey bee viruses in Europe.

The majority of studies were field studies or had a field component (91%, n = 209), which is similar to previous trends. Only 14% of studies (n = 32) were performed wholly or partly in the laboratory. A small number of studies used a modelling approach to gauge potential competitive interactions (2%, n = 4). Of field trials, 61% were correlative only and 39% included a manipulative component. For each of these categories, a similar percentage of studies indicated negative outcomes (68% and 69% respectively). Hive additions or removals were integrated into 24% of trials (n = 56). Of these, 68% suggested negative outcomes, which was nearly identical to the percentage of potentially negative outcomes found in studies that did not have a hive addition or removal (67%). Taken together, about two in three studies indicated negative impacts of managed and introduced bees in these broad categories.

### Competition between bee species

Most of the studies that investigated effects of *Apis* species (*Apis mellifera, A. cerana, A. florea*) concerned interactions with unmanaged solitary bee species (42%, n = 73), followed by interactions with *Bombus* species (21%, n = 37). The remainder covered effects on various other insects as well as one avian study. Studies that looked at the impact of managed and introduced bumblebees included effects of at least six species as well as multiple sub-species of *B. terrestris,* mostly on other *Bombus* species (60%, n = 34), followed by *Apis* species and other bees*.* Studies on the impact of other managed and introduced species of insects or bees were often generalised to insects or floral visitors and could not be sub-categorised.

The emphasis on specific bee species in competition studies were variable by region. Generally, studies of interactions with *Apis* species predominated in all regions. In regards to the disproportionate regional emphasis, much of research interest comes from regions where *Apis mellifera* or *Bombus* species have been introduced. In Oceania for example, the impacts of feral *A. mellifera* on the diverse bee fauna of Australia or the unique island fauna of the Pacific islands are of particular interest (Gilpin et al., 2014; Groom et al., 2014; Iwasaki et al., 2018; Prendergast and Ollerton, 2021; Purkiss and Lach, 2019). In studies involving *Bombus* species there were clear emphases for regions where *Bombus terrestris* has been introduced and may be competing with native bees or had introduced diseases (Japan, Argentina, Chile; Agüero et al., 2020; Nishikawa et al., 2019; Schmid-Hempel et al., 2014), or where subspecies of *B. terrestris* from commercial hives may be causing issues for local sub-species (United Kingdom; Ings et al., 2006).

As *Apis mellifera* were the most frequently studied bee, we have included a brief comparison of outcomes by bee sizes compared to *A. mellifera* (10 – 12 mm). Negative impacts were most pronounced for bees larger than *Apis mellifera* (Table 2). Of bee competition studies that identified impacted bee species for which a size could be estimated (n = 46), 48% of all studies were on larger bees (n = 22), 22% were on similar sized bees (n = 10), and 30% on small bees (n = 14). Although the more pronounced competitive effects on larger generalist bees are likely to be related to their relatively large foraging needs and high niche overlap (Henry and Rodet, 2018), the data did not allow us to discuss whether the results for large bees are due to disproportionate competition or because of taxonomic biases in the species studied. For example, smaller social bees, mostly stingless bees (Meliponini), had more even harmful/harmless outcome ratios with honey bees (1:1, n = 10). In regards to biases, a majority of high quality studies on honey bee competition are conducted on bumblebees (Wojcik et al., 2018). Bumblebees are a highly visible charismatic group of bees that are also important pollinators, and thus garner disproportionate attention. They also have shown potential competition because of high niche overlap in a variety of habitats (Goulson et al., 2002; Iwasaki et al., 2018; Thomson, 2006). The effect of managed and introduced bumblebee species showed similar patterns of biases and negative outcomes for size groups, although most studies involved other *Bombus* species. Compared to honey bee studies, bumblebee studies were mostly limited to assessing effects on other species of *Bombus* to the neglect of other bee species, reflecting the disproportionate interest in the invasive potential of *Bombus terrestris*. Thus there is the most room for improvement in research on how bumblebees affect other bees.

Evidence for competition between bee species is broadly supported to be primarily exploitative in nature in regards to floral resources, and only occasionally as physical displacement on floral resources (Cane and Tepedino, 2017; Wojcik et al., 2018). Of the studies that examined competition between bee species (n = 95), the majority investigated resource or exploitative competition (84%, n = 80), with the remainder studying interference competition on flowers (7%, n = 7). Nearly all bee competition studies involved the impacts of *Apis mellifera* or *Bombus terrestris* on other bees (Fig. 3). Of the 79 studies that examined exploitative and resource competition, a majority (68%, n = 54) reported or inferred negative outcomes. Overall, most studies measured flower visitation rates (54%, n = 51), followed by effects on foraging behaviour (26%, n = 25), and reproductive success (7%, n = 7). Of the 51 studies that investigated effects on visitation, 69% (n = 35) inferred negative outcomes. Studies involving apiary additions did not have a larger percentage of harmful outcomes than those without (61%, n = 19 vs. 65%, n = 41 respectively).

Negative outcomes were often associated with or inferred on the basis of resource overlap and associated changes/reductions in visitation in association with managed bee presence. However, changes to visitation or local floral resource use on their own may not necessarily translate to a decrease in fitness (Hudewenz and Klein, 2013; Mallinger et al., 2017; Thomson, 2006). Inferences from visitation can be informative, but expectations regarding the effects on visitation must be informed by local conditions, as a reduction in floral resources can lead to increased time spent foraging, and therefore does not necessarily lead to reduced presence on flowers (Thomson, 2006). Furthermore, even experimentally induced changes in resource use may not necessarily result in reduced fecundity if alternate floral resources are available (Paini, 2004). In addition, bee foraging behaviour may fluctuate within a day, season, or between years (Grab et al., 2017; Wratt, 1968). As a result, visitation may only reflect local conditions for that survey time. Therefore it is difficult to draw general conclusions from this substantive body of literature.

Of the nine assessments of interference competition, five outcomes suggested that *A. mellifera* aggressively displaced other bee species from flowers, while three studies found heterospecific aggression but no net displacement of one species over the other. On the whole, it has been suggested that interference competition depends on the circumstances (e.g., temperature, resource availability; Cervantes-Loreto et al., 2021; Martins, 2004), that *A. mellifera* may be slightly more aggressive than other bees (Gross et al., 2019; Pinkus-Rendon et al., 2005; Roubik, 1980), but that aggressive interference by honey bees and bumblebees is rarely observed in the field (Wojcik et al., 2018). Inconsistent conclusions for direct displacement by *Apis mellifera* and relatively few studies documenting interference competition within a large quantity of observations suggest these situations are outliers or the exception (Geslin et al., 2017; Gross and Mackay, 1998; Martins, 2004). Similarly, this may be the case for *Bombus terrestris* (Hingston et al., 1998). Exceptions include situations where males aggressively defend flower patches against most heterospecific visitors, as is the case with *Anthidium manicatum* displacing *Bombus impatiens* (Graham et al., 2019). Of the 26 studies that investigated the effects of managed or introduced species on foraging behaviour, 54 % (n = 14) found that the presence of managed or introduced bees resulted in behavioural changes. These included changes in amounts and types of pollen and/or nectar collected, time spent on flowers and peak foraging times. More in-depth observations of foraging behaviour and resource use provide specific details that give contextual information about competition, however, without an experimental assessment of the consequences for fitness, the evidence for interference competition remains weak.

The most informative studies on competition are experimental studies that directly examine effects on fitness, population size, and reproduction (Wojcik et al., 2018). Only 8% of bee competition studies (n = 7) examined reproductive success, and all but one demonstrated negative impacts. The single study that did not show a negative impact was done at temperatures that were strongly restrictive to honey bee foraging and involved a plant species from which the native *Megachile* species collected pollen, but honey bees did not (Paini and Roberts, 2005). Of the six studies that investigated the impact of the presence of hived honey bees on reproductive success of other bees, negative outcomes were found for both larger (*Bombus* spp.) and smaller species (*Exoneura asimillima, Hylaeus alcyoneus, Osmia bicornis*). Impacts of other introduced species included in reproductive studies were *Anthidium manicatum* (no impact on *Bombus impatiens*) and *Bombus terrestris dalmatinus* (negative on *B. terrestris audax*).

While general trends can be established from small groups of similar studies, the range of methods and ecologies make generalised conclusions or meta-analyses difficult. This is not surprising given the diversity in the life histories of bees, the ecosystems, and temporal differences (Wojcik et al., 2018). Thus, strong conclusions are difficult to make without insight into consequences for foraging returns, reproductive fitness, or in the longer term, population sizes. While there are significant logistical and methodological hurdles to conducting these types of studies (Wojcik et al., 2018), they represent the best possibilities for clear insights moving forward, and more such studies are needed.

### Relationships between managed/introduced bees and flora

It can be difficult to differentiate between direct effects of managed bees on plant reproduction and composition and the indirect effects via impacts on other pollinators. This reduces the utility of classifying effects into particular categories. Therefore, we limited classification of outcomes to studies that strictly involved interactions between managed bees and plants, or more narrowly described changes in seed set that are related to mutualisms or invasive mutualisms formed with introduced bees. Of the 67 studies, 58 % (n = 39) found negative or potentially negative outcomes associated with introduced bees. Neutral and positive effects were exactly split for the remainder of studies (n = 15, 13). Introduced bees (predominantly honey bees) were commonly found to have negative impacts mostly through decreased pollination services to native plants or increased seed set of invasive plant species. Positive effects are sometimes suggested when honey bees increase seed/fruit set in native plant species (Paini, 2004; Sanguinetti and Singer, 2014). However, it is unclear whether the consequential potential change in plant composition should be considered a positive contribution.

Based on their very broad diet, honey bees are sometimes called ‘supergeneralists’, as they collect and consume pollen from a very wide range of plant species (Cane and Sipes, 2006). It has been argued that this broad diet, together with their abundance and year-round presence, implies that honey bees are important connectors to plant species that otherwise would be unconnected to other pollinators (Giannini et al., 2015) and could increase the resilience of pollination networks (Giannini et al., 2015; Solé and Montoya, 2001). However, this is only one side of the coin, as interference and resource competition can decrease pollinator diversity and therefore negatively affect the resilience of pollination services (Geslin et al., 2017; Valido et al., 2019).

Honey bees have been shown to dramatically modify the structure of pollination networks, which can have major subsequent consequences for ecosystem function as visitation rates and ratios change (Tylianakis et al., 2010, 2008). These novel mutualisms have the potential to be beneficial to some species or even ecosystem function, but as mentioned may also be a net negative or have unforeseen consequences. Novel mutualisms that benefit endangered or declining species can be seen as beneficial for an ecosystem, but clearly identifying whether a novel honey bee association exists at the expense of native species can be difficult. For example, removing honey bees from islands has been suggested to benefit species of native bees and birds (Dupont et al., 2004; Kaiser-Bunbury et al., 2010; Kato et al., 1999), but control sites without honey bees are often lacking (Dupont et al., 2004). In addition, extinct or rare species may not be able to recover after local honey bee removals, making these knowledge gaps even more obtuse.

Although studies reviewed here clearly support that honey bees negatively affect plant communities via invasive mutualisms, these studies mostly examine the net reproductive output of invasive plant species. It is unclear whether large numbers of introduced or managed bee species can alter overall network properties by decreasing the connectedness and/or nestedness of pollination networks or how these changes affect bee species (Geslin et al., 2017). For assessing overall impacts on pollination networks, long-term studies that compare the effects of managed or introduced bees before and after introduction are necessary. More information on the effects of massively introduced managed species (MIMS) and their consequences for plant–pollinator interactions are discussed in the review by Geslin et al. (2017).

### The role of pathogens from managed and introduced bees

Pathogen studies are the most rapidly growing field of research involving the impacts of managed or introduced bees on other bee species. The most growth was represented in studies detecting novel pathogens or spill-over to new hosts, which is the subject of 90% of 58 studies. In all studies, these pathogens originated from either *Apis mellifera* or *Bombus* species. The majority of studies investigated the potential spread and novel detection in closely related *Apis, Bombus,* and solitary bees, followed by various hymenopterans and other floral visitors. Pathogen studies overwhelmingly demonstrated negative potential impacts (81%, n = 47) which was largely inferred from spill-over of pathogens and novel detection of disease. Most of the 52 pathogen spill-over studies focused on novel detections of a suite of related viruses (Table 1), pathogenic single-celled eukaryotes (*Apicystis bombi, Crithidia* spp., and disease-causing fungi (*Nosema* spp*., Ascosphaera apis*). Most of these are known pathogens for honey bees but their impact on other bees has not yet been thoroughly explored (Gisder and Genersch, 2017). The potential for harm to native species of hymenopterans in these studies is mostly inferred from the detection of the pathogen in the novel host. Only two studies explicitly investigated replication of the pathogen and fitness consequences for the host species.

**Table 1:** Table of pathogens/disease vectors and abbreviations used. Non-specified or identified species are abbreviated as spp.

Pathogen taxonomic group	Pathogen species	Abbreviation
Apicomplexa	*Apicystis bombi*	
Apicomplexa	*Apicystis* spp.	
Arthropod: Insect	*Aethina tumida*	
Fungi: Ascosphaeraceae	*Ascophaera* spp.	
Fungi: Ascosphaeraceae	*Ascosphaera apis*	
Fungi: Microsporidia	*Nosema bombi*	
Fungi: Microsporidia	*Nosema ceranae*	
Fungi: Microsporidia	*Nosema* spp.	
Fungi: Microsporidia	*Nosema thomsoni*	
Arthropod: Arachnida	*Acarapis* spp.	
Arthropod: Arachnida	*Locustacarus buchneri*	
Euglenozoa: Trypanosomatidae	*Crithidia bombi*	
Euglenozoa: Trypanosomatidae	*Crithidia mellificae*	
Euglenozoa: Trypanosomatidae	*Crithidia* spp.	
Euglenozoa: Trypanosomatidae	*Lotmaria passim*	
Viral	Acute bee-paralysis virus	ABPV
Viral	*Apis mellifera* filamentous virus	
Viral	Black queen cell virus	BQCV
Viral	Chronic bee paralysis virus	CBPV
Viral	Deformed wing virus	DWV
Viral	Israeli acute paralysis virus	IAPV
Viral	Kashmir bee virus	KBV
Viral	Lake Sinai virus	LSV
Viral	Macula-like virus	
Viral	Sacbrood virus	SBV
Viral	Slow bee paralysis virus	SBVP

**Table 2:** The number of studies that concluded harmful/non-harmful effects of introduced/managed bees on native pollinators, relative to their size class. Relative species size was evaluated as larger, similar, or smaller than *Apis mellifera*, the most commonly studied competitor. Larger bees were larger than honeybees (>12 mm body length) and generally included *Bombus* and *Xylocopa*. Similar sized bees (10 – 12 mm) included *Osmia bicornis* and *Bombus occidentalis*, and smaller bees (<10 mm body length) were species of *Augochloropsis, Exoneura asimillima, Hylaeus alcyoneus, Perdita meconis, Megachile rotundata, Apis cerana, Apis florea,* and stingless bees (Meliponini).

Relative species size	No harm	Harm	Total	Percent harm
Larger	5	17	**22**	77
Similar	5	5	**10**	50
Smaller	6	8	**14**	57
**Grand Total**	**16**	**30**	**46**	**65**

Studies on bumblebees examined the potential for introduced or managed bumblebees to spread pathogens to native species or populations, a situation which may be a significant contributor to ongoing declines in native bumblebees (Otterstatter and Thomson, 2008). In South America there is particular concern regarding the potential of *B. terrestris* to spread pathogens among native *Bombus* populations, especially endangered populations of the native bumblebee *B. dahlbomii* (Arbetman et al., 2013; Arismendi et al., 2021). In Japan, there is a similar concern that introduced diseases will spread throughout diverse communities of native bumblebees (Kojima et al., 2011; Niwa, S., Iwano, H., Asada, S. I., Matsumura, M., Goka, 2004). Honey bee apiaries and managed bumblebee colonies can contribute to the spread of these diseases by situating them in areas where they are not usually found and/or by acting as centres of disease replication that increase local disease densities and subsequent likelihood of spill-over (Graystock et al., 2016; Ravoet et al., 2014).

Overall, a large proportion of research has been conducted on charismatic species, particularly bumblebees (*Bombus* spp.), while, aside from novel detection or spill-over, there is a dearth of information about the impacts of pathogens on solitary species. The understanding of whether and how novel diseases will impact solitary or other non-managed native bee communities is limited and pertains only to a few managed solitary species (e.g., *Megachile rotundata* and *Osmia* spp.). Novel diseases that infect native bees can subsequently become endemic within pollinator communities (Ravoet et al., 2014). The growing demand for managed pollinators for the agricultural sector further increases the risks of increased disease prevalence due to travel with hives and unnaturally high densities of hosts (Gisder and Genersch, 2017). Therefore, it is highly likely that the risks of novel pathogens and subsequent spill-over in pollinator populations will only grow in the future.

It has been known for quite some time that bee pathogens can infect and replicate in closely related species and the growth in pathogen detection studies reflects an increased awareness of this issue. This has been partly caused by an increased potential for novel disease transmission due to global trafficking of bee species, and partly by the increasing ability to detect and monitor these patterns (Levitt et al., 2013; Potts et al., 2010; Singh et al., 2010). It is now abundantly clear that previously novel discoveries of cross-species or higher order cross-susceptibility (disease transmission from wasps and ants) are caused by widespread disease susceptibility and that there is ongoing and global transmission (Arbetman et al., 2013; Fürst et al., 2014; Genersch et al., 2006; Graystock et al., 2016).

The potential for harm caused by these novel pathogens to native bee communities, and the extent to which they will become endemic, largely remains to be determined (Tehel et al., 2020). Novel pathogen introduction may often not result in spread or in strong pathogenicity within a community (Dolezal et al., 2016). Pathogen prevalence may only be limited to high local densities of managed species (Pritchard et al., 2021). On the other hand, there are examples of novel pathogens becoming established and having severe consequences in naïve populations, as is suspected with *B. dahlbomii* and the protozoan *Apicystis bombi* (Arbetman et al., 2013), and has been seen in the establishment of the microsporidian *Nosema ceranae* and *Apis mellifera* (Klee et al., 2007).

In conclusion, it is clear that there are severe risks from the global trade and movement of managed pollinators to native pollinators (Geslin et al., 2017; Graystock et al., 2016) with strong biosecurity implications, but further studies are needed to assess the potential pathogenicity of nearly all pathogens for unmanaged bee species.

### Impacts of managed and introduced bees on vertebrates

Research on interference or food competition with nectar feeding birds, or displacement of birds on flowers (Paton, 1996), were not found using our search criteria. However, our search terms returned one study that examined the impacts of managed honey bees on nesting habitat of birds, where removal experiments have been shown to significantly increase bird occupancy and breeding (Pacífico et al., 2020). This likely represents a marked deficit in research on the effects of managed and introduced bees on vertebrates, both in terms of resource competition and for nesting competition.

### Modelling specific studies

Of newer studies, five conducted model-based investigations without direct observations of bee behaviour to estimate the impacts of introduced bees on pollination networks, potential impacts on other bees from historical records, projected resource use, network analyses, or spatial overlap. These models or estimates were primarily designed to identify the factors behind indirect competition (Cane and Tepedino, 2017), how introduced species alter floral interactions within a community (e.g., pollination networks, evolutionary consequences of floral network changes; Milner et al., 2020; Rasmussen et al., 2021), or habitat suitability for invasion success (Naeem et al., 2018). We are aware that these are not the only studies that examine these topics (Russo et al., 2019; Valdovinos et al., 2018), but unfortunately not all types of studies suited criteria for inclusion in discussion. All models which examined specific competitive effects found the potential for harm.

## Conclusions

The recent increase in research on competition between managed/introduced and native bees has likely been driven by concerns for native bee declines and their importance to agricultural production (Dicks et al., 2021; Potts et al., 2016, 2010). While similar concerns may be involved in the recent increases in research on pathogen transmission between bee species, it is also possible that Varroa-related spread of viruses and colony collapse disorder among honey bees have contributed (Fürst et al., 2014; McMahon et al., 2015; Pirk et al., 2017).

Previous reviews have pointed out the difficulties in definitively summarising the relative effect of managed and introduced bee species on wild bees, caused by a myriad of dependent variables, confounding factors, and mixed results (Goulson, 2003; Mallinger et al., 2017; Paini, 2004; Wojcik et al., 2018). A meta-analysis of effect sizes would be preferred over general classifications such as harm or no harm observed. However, this requires the availability of numerous controlled experimental studies to assess impacts, such as replicated assessment of impact at different hive densities (Lindström et al., 2016; Roubik and Wolda, 2001). There is as yet a clear shortage of such studies.

Overall, two out of three study outcomes indicated negative effects, while eight out of nine experimental studies that considered fitness effects on native bees found significant negative effects (Wojcik et al., 2018). This discrepancy further underscores the urgent need for replicated experimental research on the effects of managed and introduced bees on fitness of native species, as noted in earlier reviews (Goulson, 2003; Mallinger et al., 2017; Paini, 2004; Wojcik et al., 2018). However, the small number of experimental studies provide relatively strong evidence for negative impacts, and they are supported by a large and increasing number of observational or correlative studies. Likewise, we concur with Geslin et al. (2017) in concluding that MIMS can severely disrupt local pollination networks and form strong invasive mutualisms that negatively affect native ecosystems. Finally, our review summarises a near-consensus on the risks of pathogen transmission from managed and introduced bees to wild populations (Fürst et al., 2014; Pirk et al., 2017). However, while the consequences from pathogen spill-over have largely been inferred as negative, for most species, there still is a lack of understanding in subsequent fitness consequences of introducing novel pathogens into wild populations (Tehel et al., 2020). Regardless, the increasing prevalence of novel bee diseases and widespread transport of managed hives warrant caution (Pirk et al., 2017).

Multiple actions are needed to prevent further damage from managed bees to native ecosystems. For example, assessments of the risk involved in introducing novel pollinator species that include introduction of pathogens, feral establishment, and potential damage caused to native ecosystems should be undertaken. In addition, further protection for wild bees in both crop pollination systems and natural ecosystems is needed (Iwasaki and Hogendoorn, 2021b; Table 1). In agricultural landscapes, increased floral support can help to maintain diverse and resilient crop pollination, including support for wild honey bees in areas where they are native (Dicks et al., 2015; Evans et al., 2018; Pardee and Philpott, 2014; Requier et al., 2019). To protect native ecosystems, apiary sites should not be allowed in conservation areas. While bee declines, and/or the importance of honey bees as crop pollinators are often used as arguments to support access to conservation areas for honey production, these arguments are tenuous for two reasons. Firstly, managed bees, in particular honey bees, are livestock, and despite winter losses, including from colony collapse disorder, hive numbers have been increasing and these species are therefore not in decline (Aizen and Harder, 2009; Osterman et al., 2021). Secondly, honey may be an essential food item for honey bees, but it is not essential for humans. In addition, in regions where managed bees have become feral and problematic for native species (e.g., *B. terrestris* in Chile and Japan, *A. mellifera* in Australia), research is required how to control these invasive pest species and limit their impact in the most vulnerable habitats. Finally, bee-washing, a type of greenwashing that misinforms the public to embrace beekeeping and consumption of honey bee products under the guise of native bee conservation, has become rampant in the last decade. Even when well intentioned, this issue has the potential to pivot environmental legislation and action away from evidence-based decision-making that assists declining species (Colla, 2022).

## CRediT authorship contribution statement

**Jay M. Iwasaki:** Conceptualization, Writing – review & editing, Data curation, Formal analysis. **Katja Hogendoorn:** Conceptualization, Writing – review & editing, Data curation, Formal analysis.

## Declaration of Competing Interest

The authors declare that they have no known competing financial interests or personal relationships that could have appeared to influence the work reported in this paper.

## Appendix Supplementary materials

Image, application 1

Image, application 2
